# Reduced miR-200b and miR-200c expression contributes to abnormal hepatic lipid accumulation by stimulating JUN expression and activating the transcription of *srebp1*

**DOI:** 10.18632/oncotarget.9183

**Published:** 2016-05-05

**Authors:** Jun Guo, Weiwei Fang, Libo Sun, Yonggang Lu, Lin Dou, Xiuqing Huang, Mingxiao Sun, Cheng Pang, Jing Qu, Guanghui Liu, Jian Li

**Affiliations:** ^1^ The Key Laboratory of Geriatrics, Beijing Hospital and Beijing Institute of Geriatrics, Ministry of Health, Beijing 100730, China; ^2^ Graduate School of Peking Union Medical College and Chinese Academy of Medical Sciences, Beijing 100730, China; ^3^ National Laboratory of Biomacromolecules, Institute of Biophysics, University of Chinese Academy of Sciences, Chinese Academy of Sciences, Beijing 100101, China; ^4^ Department of Hepatobiliay Surgery and You-An Liver Transplantation Center, Beijing You-An Hospital, Capital Medical University, Beijing 100069, China; ^5^ State Key Laboratory of Stem Cell and Reproductive Biology, Institute of Zoology, Chinese Academy of Sciences, Beijing 100101, China; ^6^ University of Chinese Academy of Sciences, Beijing 100049, China

**Keywords:** miR-200b, miR-200c, lipogenesis, JUN, SREBP1

## Abstract

Previous studies indicated that miR-200s participated in IL-6-induced hepatic insulin resistance. However, the role of miR-200s in hepatic lipid accumulation has not been elucidated. Here we found that miR-200b and miR-200c were reduced in the steatotic livers of mice fed a high-fat diet (HFD) and patients with nonalcoholic fatty liver disease. This down-regulation was accompanied by an increase in the expression of lipogenic proteins such as sterol regulatory element-binding protein 1 (SREBP1) and fatty acid synthase (FAS). The suppression of miR-200b and miR-200c in Hep1-6 and NCTC1469 hepatocytes enhanced intracellular triglyceride levels, which were associated with increased SREBP-1 and FAS protein levels. In contrast, the over-expression of miR-200b and miR-200c suppressed lipid accumulation and reduced the expression of SREBP1 and FAS in Hep1-6 and NCTC1469 cells transfected with miR-200b or miR-200c mimics. Importantly, the up-regulation of miR-200b and miR-200c could reverse oleic acid/palmitic acid-induced lipid accumulation in hepatocytes. A luciferase reporter assay identified that miR-200b and miR-200c could directly bind the 3′UTR of *jun*. JUN activated the transcription of *srebp1* to increase lipid accumulation. The data also demonstrated that increased miR-200b and miR-200c expression might be associated with sitagliptin-reduced hepatic lipid accumulation in mice fed a HFD. These findings suggest, for the first time, that reduced miR-200b and miR-200c expression contributes to abnormal hepatic lipid accumulation by stimulating JUN expression and activating the transcription of *srebp1*.

## INTRODUCTION

Obesity is a global health problem that causes a series of pathological disorders including nonalcoholic fatty liver disease (NAFLD), type 2 diabetes and cardiovascular disease [1–3]. Abnormal lipid accumulation in the liver may result in hepatic steatosis and nonalcoholic steatohepatitis (NASH) [4, 5]. Great advances have been made in defining the pathogenesis of NAFLD. However, the specific molecular mechanism responsible for the disease is still poorly understood.

Liver lipid homeostasis is correlated with different processes including the uptake, synthesis, storage and secretion of lipids [6]. As major transcription factors, sterol regulatory element-binding proteins (SREBPs) have been reported to widely regulate lipogenic gene expression [7]. The SREBP family includes SREBP-1a, SREBP-1c and SREBP-2, all of which are transcribed from two distinct genes, *srebp1 and srebp2*. Two alternatively spliced transcripts, *srebp-1a* and *srebp-1c*, are expressed from various promoters [8]. SREBP-1c mainly stimulates proteins involved in fatty acid metabolism such as fatty acid synthase (FAS) [9, 10]. In comparison, SREBP-2 and SREBP-1a mainly participate in cholesterol metabolism [11].

MicroRNAs (miRs) are small, non-coding RNAs that are important post-transcriptional regulators of gene expression. By binding to the 3′ untranslated region (3′UTR) of their target genes, they globally repress gene expression [14]. miRs have been shown to be involved in a wide variety of physiological and psychological processes [15, 16]. miR-34a was increased in patients with NAFLD and in mice fed a high-fat diet (FHD-fed mice) [17]. miR-33a and miR-33b were found to globally regulate lipid metabolism by targeting numerous lipid metabolism-associated genes including *abca1, crot, cpt1a, hadhb* and *ampk*α [19]. The miR-200 family, including miR-200a, miR-200b, miR-200c, miR-141 and miR-429, has been reported to be dysregulated in several types of cancers including gastric cancer, breast cancer and bladder cancer [20–22]. In a previous study, we demonstrated that the reduction of miR-200a, miR-200b and miR-200c in hepatocytes contributed to IL-induced insulin resistance [23]. However, the role of the miR-200 family in hepatic lipid metabolism remains unknown. The aim of this study was to explore the potential role of the miR-200 family in the regulation of hepatic lipid metabolism. The findings suggest that the reduced expression of miR-200b and miR-200c participated in abnormal hepatic lipid accumulation by stimulating JUN expression and activating the transcription of *srebp1*.

## RESULTS

### The levels of miR-200b and miR-200c are reduced in the steatotic livers of HFD-fed mice and NAFLD patients

**Figure 1:**
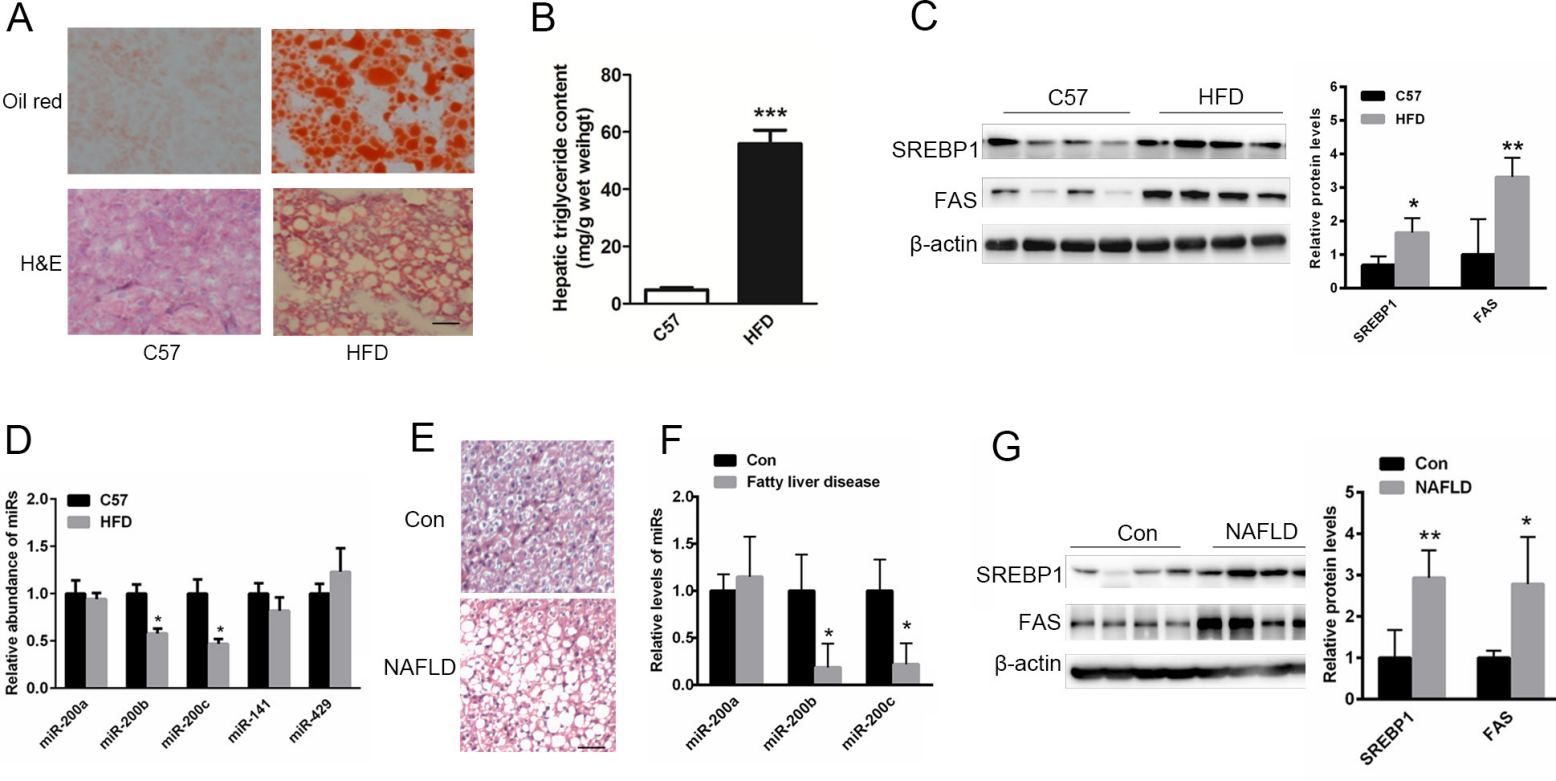
The levels of miR-200b and miR-200c are reduced in the steatotic livers of NAFLD patients and mice fed a HFD (**A**) Oil red O and H&E staining of the livers of HFD-fed mice. (**B**) The measurement of triglyceride levels in the livers of HFD-fed mice. (**C**) Western blots showing the expression of the lipogenic proteins SREBP1 and FAS. (**D**, **F**) Real-time reverse-transcription PCR showing the relative expression patterns of miR-200 family members including miR-200b, miR-200c, miR-200a, miR-141 and miR-429 in the steatotic livers of HFD-fed mice (*n* = 5) or in the livers of NAFLD patients and healthy subjects (*n* = 11). (**E**) H&E staining of the livers of NAFLD patients. (**G**) Western blots showing the expression of SREBP1 and FAS in the livers of NAFLD patients. The data represent the mean ± SEM. *P < 0.05 and **P < 0.01 versus the control. The bar represents 25 μm.

Oil red O and H&E staining showed abnormal lipid accumulation in the livers of mice fed a HFD (Figure 1A). The livers of HFD-fed mice displayed excessive amounts of triglycerides (Figure 1B). Moreover, the expression of lipogenic genes including *srebp1* and *fas* was significantly increased in the livers of HFD-fed mice (Figure 1C). To identify the potential role of the miR-200 family in lipid metabolism, relative expression patterns were analyzed in the steatotic livers of HFD-fed mice and NAFLD patients. As shown in Figure 1D, the levels of miR-200b and miR-200c, but not the levels of other members of the miR-200 family including miR-200a, miR-141 and miR-429, were obviously reduced in the livers of HFD-fed mice. As shown in Table 1, the age and gender distribution were similar between the healthy controls and NAFLD patients. Characteristics such as BMI, waist circumference and triglyceride levels were significantly higher in the NAFLD patients compared with the healthy controls. H&E staining showed that the cytoplasm of the NAFLD patient hepatocytes was filled with lipid droplets (Figure 1E). Importantly, the expression of miR-200b and miR-200c was suppressed in the livers of NAFLD patients (Figure 1F), and the levels of lipogenic proteins such as SREBP1 and FAS were elevated compared with the healthy controls (Figure 1G). These data suggest that miR-200b and miR-200c may be involved in hepatic lipogenesis.

**Table 1:** Clinical and biochemical characteristics of healthy controls and patients with nonalcoholic fatty liver disease1 *n* (%)

Characteristic	Control (*n* = 10)	NAFLD (*n* = 10)	*P* value
Gender (males/females)	6/5	6/5	—
Age (yr)	43.0 ± 8.3	42.5 ± 9.8	0.906048
BMI (kg/m^2^)	23.0 ± 4.9	27.0 ± 2.9	0.029506
Smoking	no	no	—
Waist circumference (cm)	77.4 ± 9.6	93.1 ± 8.6	0.001175
Diabetes Mellitus	no	no	—
Metabolic syndrome	no	no	—
Hypertension	no	no	—
Systolic blood pressure (mmHg)	108.2 ± 7.5	114.9 ± 7.5	0.045535
Diastolic blood pressure (mmHg)	73.3 ± 5.5	76.8 ± 5.5	0.138200
AST median (min-max, U/L)	16.7 (13–24)	18.1 (12–26)	0.410204
ALT median(min-max, U/L)	13.8 (8–23)	21.7(11-38)	0.024995
Total cholesterol (mmol/L)	4.5 ± 0.4	5.0 ± 1.1	0.129345
HDL-cholesterol (mmol/L)	1.5 ± 0.3	1.3 ± 0.2	0.090324
LDL-cholesterol (mmol/L)	2.0 ± 0.7	2.9 ± 1.3	0.074496
Triglycerides (mmol/L)	0.8 ± 0.3	2.3 ± 1.7	0.007739

### Reduced miR-200b and miR-200c expression contributes to abnormal lipid accumulation in Hep1-6 and NCTC1469 cells

**Figure 2:**
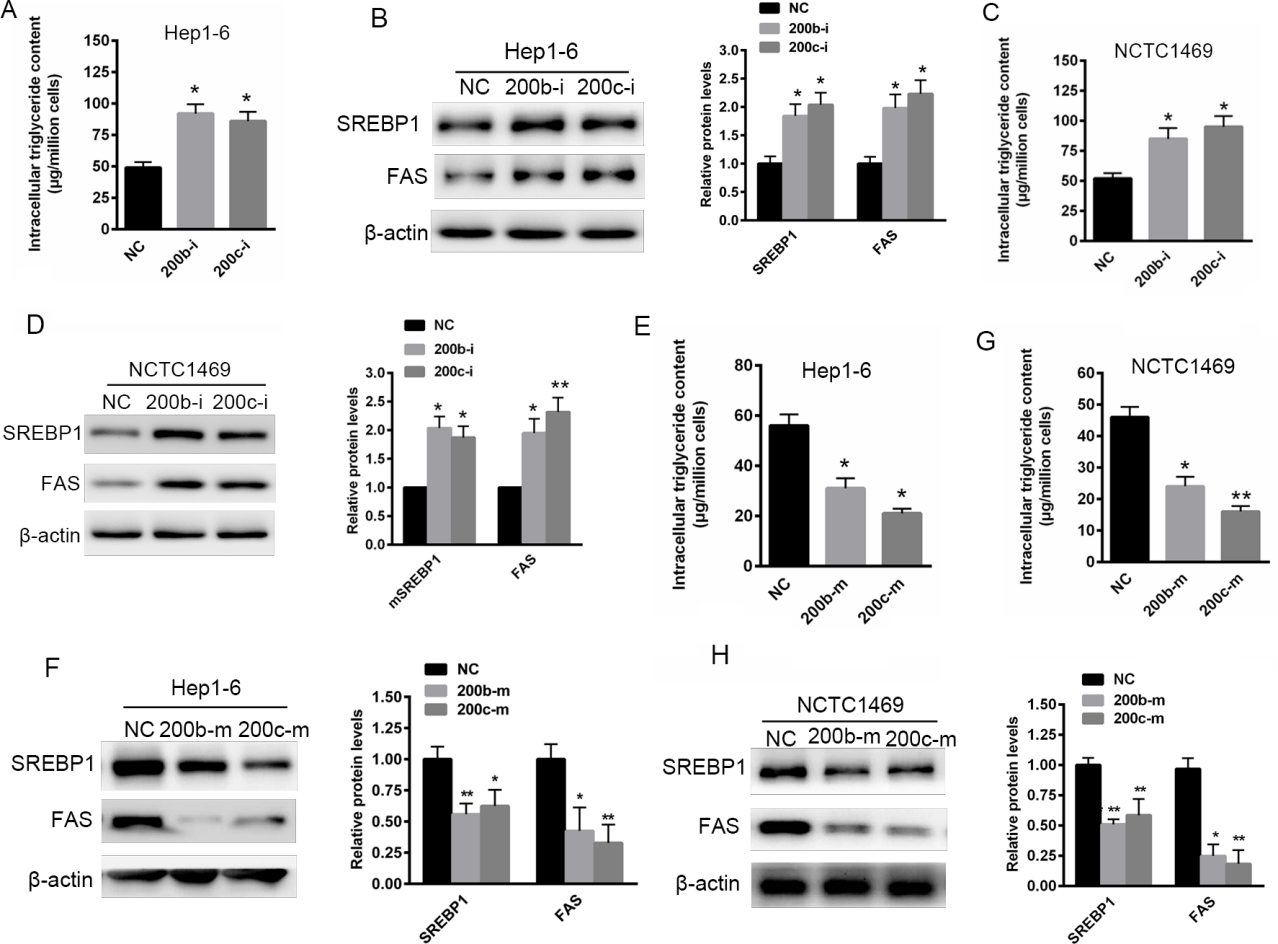
Reduced miR-200b and miR-200c expression contributes to abnormal lipid accumulation in Hep1-6 and NCTC1469 cells (**A**, **B**) The measurement of triglyceride levels in Hep1-6 and NCTC1469 murine liver cells transfected with miR-200b and miR-200c inhibitors. (**C**, **D**) Western blots showing the expression of SREBP1 and FAS in Hep1-6 and NCTC1469 cells transfected with miR-200b and miR-200c inhibitors. (**E**, **F**) The measurement of triglyceride levels in Hep1-6 and NCTC1469 cells transfected with miR-200b and miR-200c mimics. (**G**, **H**) Western blots showing the expression of SREBP1 and FAS in Hep1-6 and NCTC1469 cells transfected with miR-200b and miR-200c mimics. The data represent the mean ± SEM of three independent experiments. *P < 0.05 and **P < 0.01 versus the control.

To determine whether miR-200b and miR-200c are involved in abnormal hepatic lipid accumulation, the expression of miR-200b and miR-200c was suppressed in two murine liver cell lines, Hep1-6 and NCTC1469, by transfection with miR-200b and miR-200c inhibitors. As shown in Figure 2A and 2B, the transfection of both Hep1-6 and NCTC1469 cells with miR-200b and miR-200c inhibitors resulted in a significant increase in intracellular triglyceride levels. This in turn was associated with elevated SREBP-1 and FAS protein levels (Figure 2C and 2D). This finding suggests that reduced miR-200b and miR-200c expression might contribute to abnormal lipid accumulation in hepatocytes. In contrast, the over-expression of miR-200b and miR-200c significantly reduced lipid accumulation in Hep1-6 and NCTC1469 cells transfected with miR-200b and miR-200c mimics (Figure 2E and 2F). This was accompanied by decreased SREBP1 and FAS levels (Figure 2G and 2H), indicating that miR-200b and miR-200c mimics have a suppressive role in lipid accumulation.

### The over-expression of miR-200b and miR-200c reverses oleic acid/palmitic acid-induced lipid accumulation in hepatocytes

**Figure 3:**
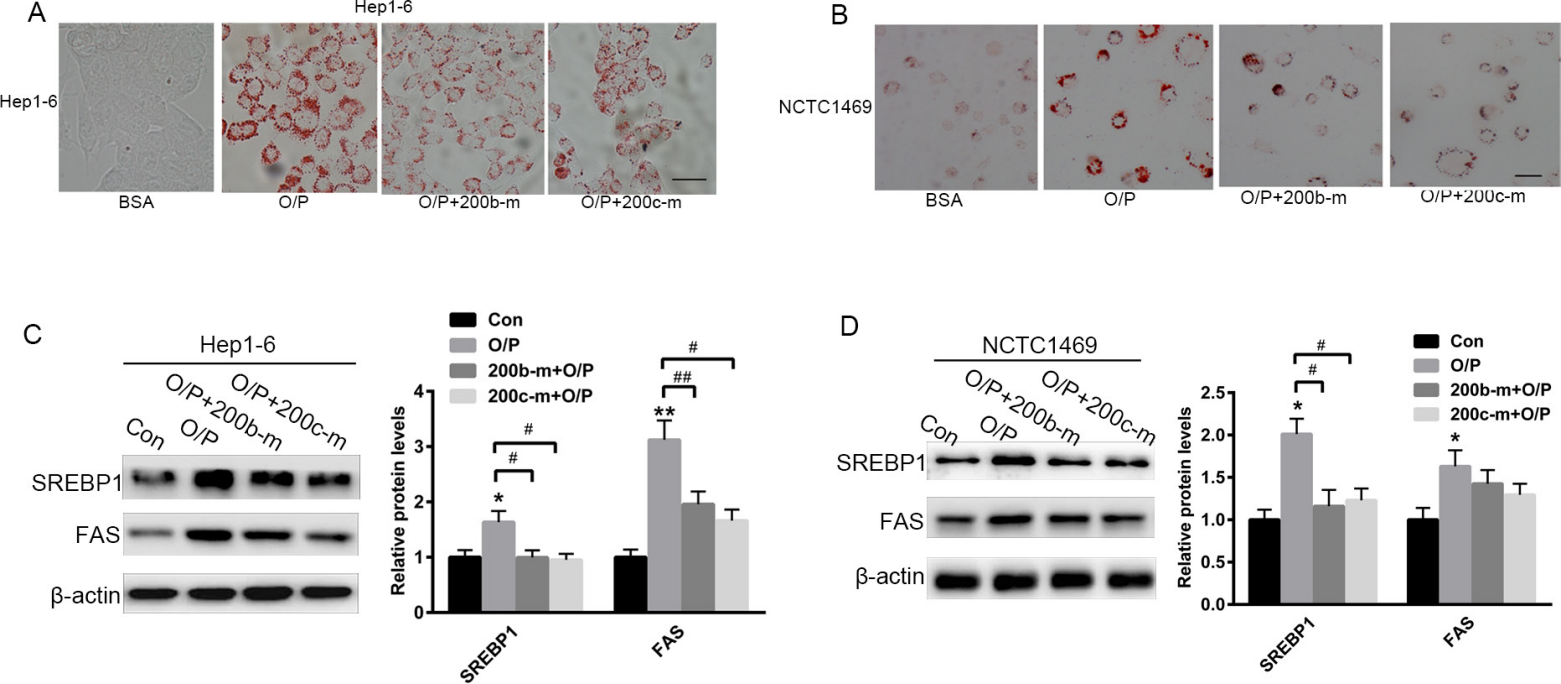
The over-expression of miR-200b and miR-200c reverses oleic acid/palmitic acid-induced lipid accumulation in hepatocytes (**A**, **B**) Oil red O staining of Hep1-6 and NCTC1469 cells pre-treated with a mixture of oleic acid/palmitic acid (2:1, M/M) for 24 h. (**C**, **D**) Western blots showing the expression of SREBP1 and FAS in Hep1-6 and NCTC1469 cells pre-treated with a mixture of oleic acid/palmitic acid (2:1, M/M) for 24 h and then transfected with miR-200b and miR-200c mimics. The data represent the mean ± SEM of three independent experiments. *P < 0.05 and **P < 0.01 versus the control; ^#^*P* < 0.05 and ^##^*P* < 0.01 versus O/P. The bar represents 10 μm.

To further investigate the suppressive role of miR-200b and miR-200c mimics in lipid accumulation, Hep1-6 and NCTC1469 cells were pre-treated with a mixture of oleic acid and palmitic acid (2:1, M/M) for 24 h. Oil red O staining revealed that pre-treatment with oleic acid/palmitic acid (O/P) significantly promoted lipid accumulation in Hep1-6 and NCTC1469 cells (Figure 3A and 3B). Interestingly, the transfection of both Hep1-6 and NCTC1469 cells with miR-200b and miR-200c mimics partially reversed the formation of the O/P-induced lipid droplets (Figure 3A and 3B) and the elevation of SREBP1 and FAS levels (Figure 3C and 3D).

### *jun* is a target gene of miR-200b and miR-200c

**Figure 4:**
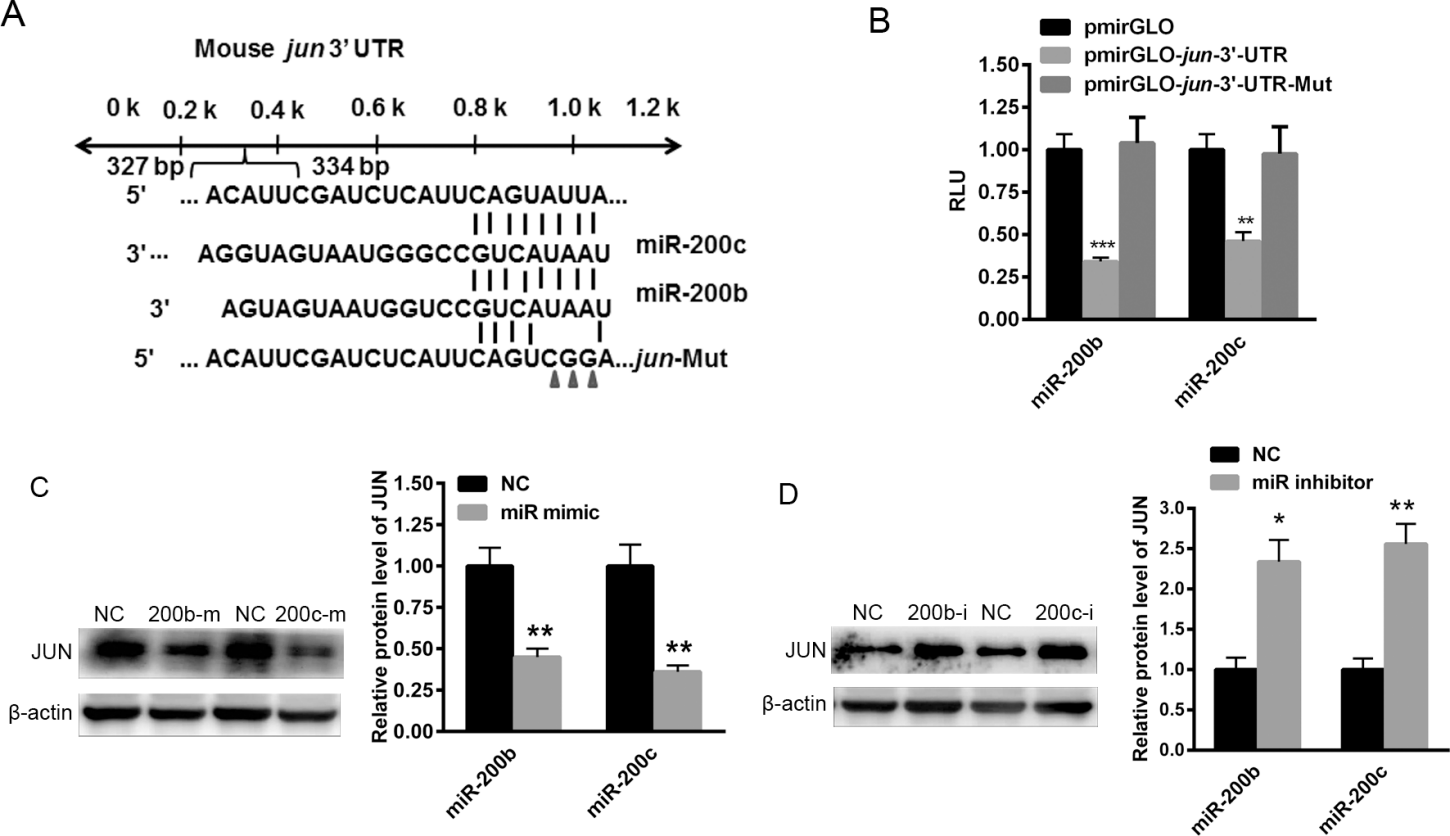
miR-200b and miR-200c bind to the 3′UTR of *jun* (**A**) TargetScan-predicted miR-200b and miR-200c bind to the 3′UTR of *jun*. (**B**) The luciferase reporter assay used to determine the effects of miR-200b and miR-200c on the relative luciferase activity of pmirGLO-*jun*-3′UTR. (**C**) Western blots showing the expression of the JUN protein in Hep1-6 cells transfected with miR-200b and miR-200c mimics. (**D**) Western blots showing the expression of the JUN protein in Hep1-6 cells transfected with miR-200b and miR-200c inhibitors. The data represent the mean ± SEM of three independent experiments. *P < 0.05 and **P < 0.01 versus the control.

To explore the specific mechanism by which miR-200b and miR-200c regulate lipogenesis in hepatocytes, miR-200b and miR-200c target genes were identified through the bioinformatics websites miRBase, TargetScan and RNA22. Unfortunately, no target genes directly involved in lipid metabolism were identified. Instead, a transcription factor, *jun*, was predicted to be a target gene of miR-200b and miR-200c (Figure 4A). To verify this finding, the 3′UTR of *jun* was cloned into the pmirGLO plasmid. As shown in Figure 4B, the miR-200b and miR-200c mimics significantly reduced the relative luciferase activity of pmirGLO-*jun*-3′UTR, indicating a direct binding. Furthermore, the mutant of *jun* 3′UTR was inserted into the pmirGLO plasmid, but overexpression of miR-200b and miR-200c could not decrease the relative luciferase activity of pmirGLO-*jun*-3′UTR-Mut (Figure 4B). Western blotting showed that the over-expression of miR-200b and miR-200c obviously reduced the level of JUN protein (Figure 4C), while the inhibition of miR-200b and miR-200c significantly elevated the expression of JUN in Hep1-6 cells (Figure 4D), demonstrating that *jun* is a target gene of miR-200b and miR-200c.

### JUN stimulates lipid accumulation by activating the transcription of *srebp1*

**Figure 5:**
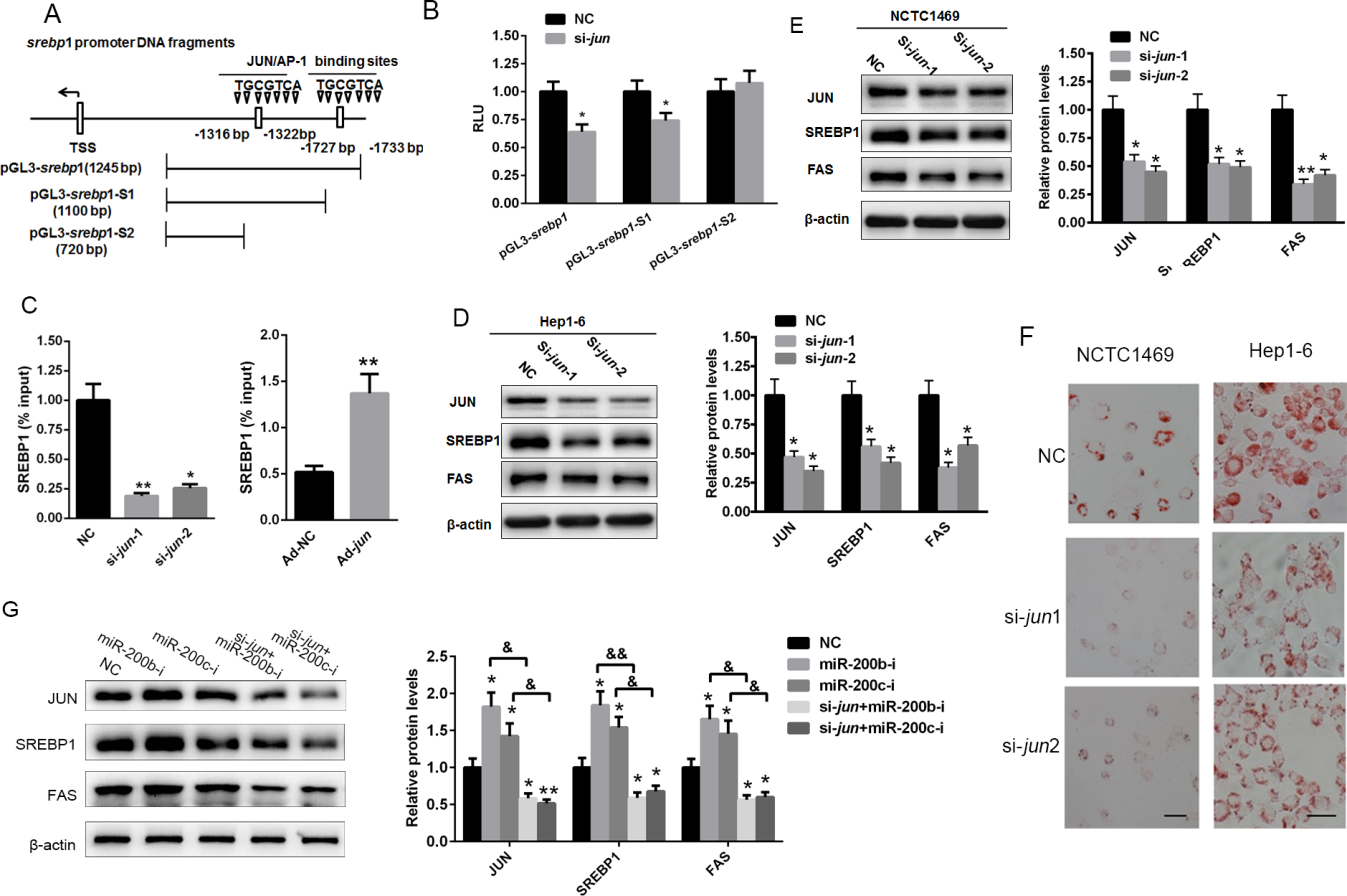
JUN activates lipid accumulation by activating the transcription of *srebp1* (**A**) Schematic analysis of the potential JUN binding sites in the promoter region of murine *srebp1* gene. (**B**) Results of the luciferase reporter assay conducted in HEK293T cells co-transfected with siRNA specifically inhibiting *jun* (si- *jun*-1 or si- *jun*-2) and a luciferase reporter plasmid containing the promoters of either *srebp1* (pGL3-*srebp1*), *srebp1*-S1 (pGL3- *srebp1*-S1) or *srebp1*-S2 (pGL3- *srebp1*-S2). (**C**) A *srebp1* ChIP assay was performed in Hep1-6 cells transfected with si- *jun*/NC (upper panel) or ad- *jun*/N C (lower panel) for 48 h. (**D**, **E**) Western blot analysis of SREBP1 and FAS expression in Hep1-6 and NCTC1469 cells transfected with si-*jun*-1 or si-*jun*-2. (**F**) Oil red O staining of Hep1-6 and NCTC1469 cells transfected with si-*jun*-1 or si-*jun*-2. (**G**) Western blot analysis of SREBP1 and FAS protein levels in Hep1-6 cells co-transfected with si-*jun*-1 and either the miR-200b inhibitor or the miR-200c inhibitor. The data represent the mean ± SEM of three independent experiments. *P < 0.05 and **P < 0.01 versus the control; ^&^*P* < 0.05 and ^&&^*P* < 0.01 versus the miR-200b inhibitor or the miR-200c inhibitor. The bar represents 10 μm.

As a transcription factor, JUN participates in cell proliferation, cell survival, apoptosis and tumorigenesis by activating gene expression [24, 25]. In the present study, two putative JUN binding sites were identified in the promoter region of the mouse *srebp1* gene. To confirm the potential binding sites, the regions of the *srebp1* promoter containing both of the binding sites, one of the binding sites (pGL3-*srebp1*-S1) or neither of the binding sites (pGL3-*srebp1*-S2) were cloned into the pGL3 vector (Figure 5A). HEK293 cells were then transfected with siRNA specifically targeting *jun* (si-*jun*-1 or si- *jun*-2) along with the luciferase reporter plasmids containing *srebp1*, *srebp1*-S1 or *srebp1*-S2. The results indicated that the knockdown of *jun* decreased the relative luciferase activity of pGL3-*srebp1* and pGL3-*srebp1*-S1, but had no effect on pGL3-*srebp1*-S2 (Figure 5B). Furthermore, a ChIP assay revealed that the knockdown of *jun* in Hep1-6 cells reduced the binding between JUN and the *srebp1* promoter, while the over-expression of JUN enhanced it (Figure 5C). The knockdown of *jun* reduced SREBP1 and FAS protein levels (Figure 5D and 5E) as well as lipid accumulation (Figure 5F) in both Hep1-6 and NCTC1469 cells. More importantly, the knockdown of *jun* could partially ameliorate the miR-200b and miR-200c inhibition-induced increased SREBP1 and FAS levels in Hep1-6 cells (Figure 5G). Taken together, these data suggest that miR-200b and miR-200c are involved in lipogenesis through stimulation of JUN expression and the subsequent activation of *srebp1* transcription.

### The up-regulation of miR-200b and miR-200c expression in the liver prevents hepatic lipid accumulation in HFD-fed mice

**Figure 6:**
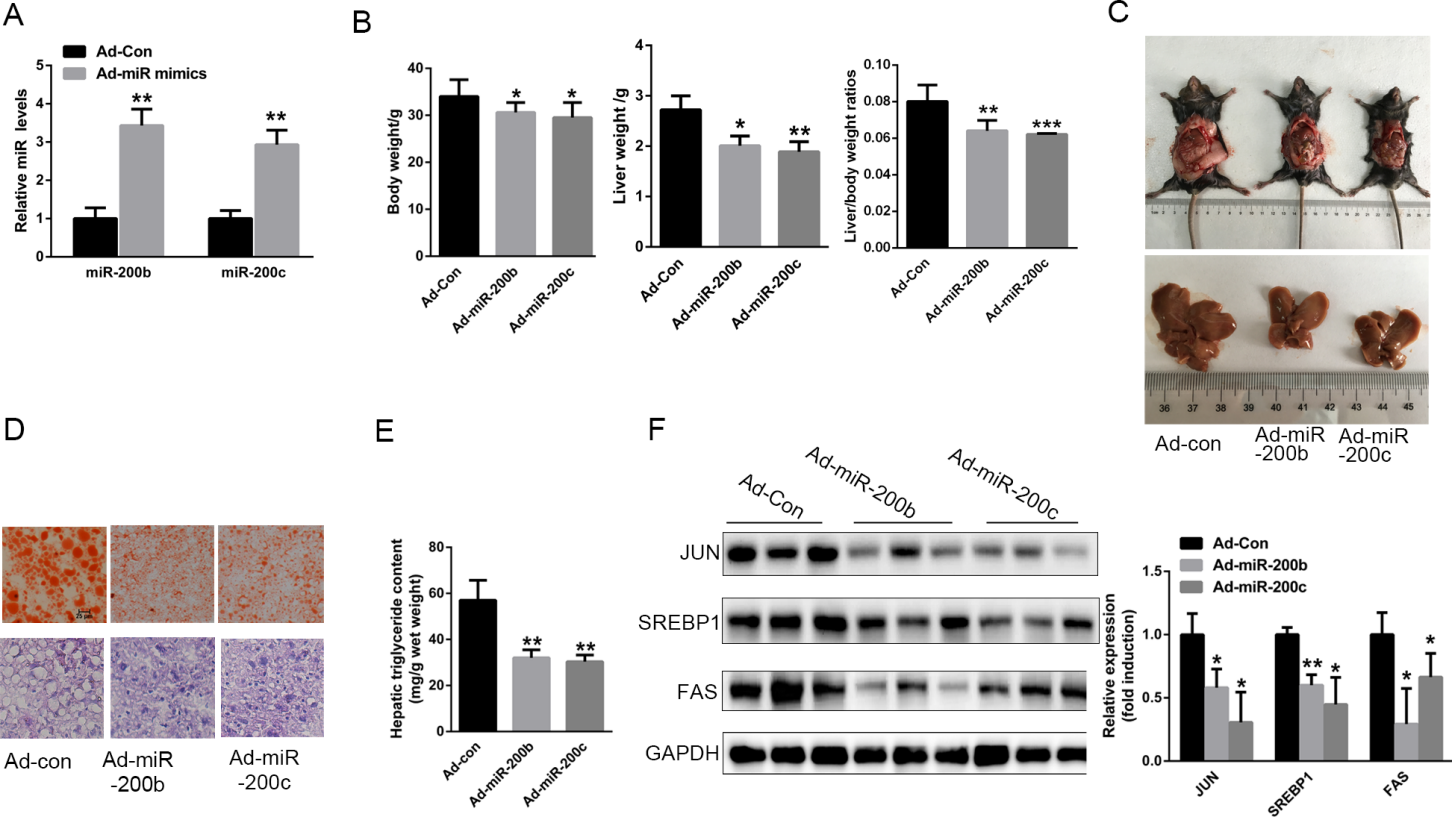
The up-regulation of miR-200b and miR-200c expression in the liver prevents hepatic lipid accumulation in mice fed a HFD Six-to-eight-week-old male C57BL/6J mice fed a HFD for 8 weeks were injected with Ad-200b, Ad-200c or Ad-con through the tail vein and sacrificed 7 days later. (**A**) Levels of hepatic miR-200b and miR-200c. (**B**, **C**) Body weight, liver weight and liver weight-to-body weight ratio. (**D**) H&E and Oil Red O staining of frozen liver sections. (**E**) Hepatic triglyceride content. (**F**) Western blots showing the hepatic expression of JUN, SREBP1 and FAS. The data represent the mean ± SEM, *n* = 6 mice. **P < 0.01 and ***P < 0.001 versus the control.

To determine whether miR-200b and miR-200c are involved in hepatic lipid accumulation *in vivo*, miR-200b and miR-200c were over-expressed in the livers of HFD-fed mice through the use of recombinant adenovirus expressing miR-200b and miR-200c mimics (Ad-miR-200b & Ad-miR-200c), respectively. Seven days after administration of Ad-miR-200b and Ad-miR-200c to the mice by tail vein injection, the hepatic levels of miR-200b and miR-200c were elevated by 3.43- and 2.93-fold, respectively (Figure 6A). More importantly, this enhancement of hepatic miR-200b and miR-200c expression led to a significant reduction in liver weight and in the liver weight-to-body weight ratio (Figure 6B, 6C). H&E and Oil Red O staining revealed decreased hepatic lipid deposition in these animals (Figure 6D). Of note, the hepatic triglyceride content was significantly reduced (Figure 6E), and this was associated with decreased levels of JUN, SREBP-1 and FAS protein in the liver (Figure 6F). These results suggest that the up-regulation of miR-200b and miR-200c expression in the liver may prevent the accumulation of hepatic triglycerides in HFD-fed mice by suppressing JUN-SREBP1-stimulated lipogenesis.

### Elevated miR-200b and miR-200c expression is associated with sitagliptin-reduced hepatic lipid accumulation in HFD-fed mice

**Figure 7:**
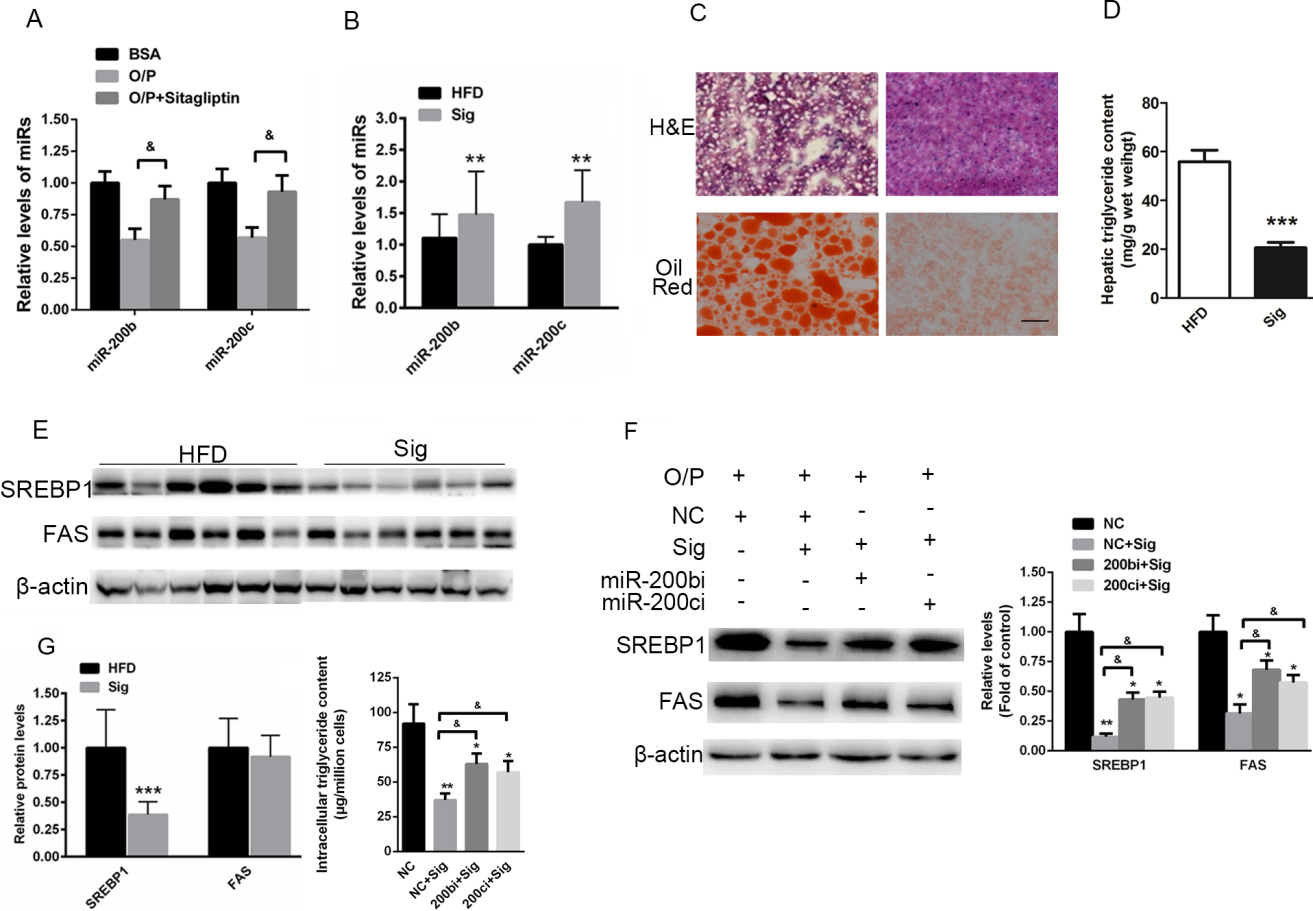
Elevated miR-200b and miR-200c expression is associated with sitagliptin-reduced hepatic lipid accumulation in mice fed a HFD (**A**) Real-time reverse-transcription PCR analysis of miR-200b and miR-200c expression in Hep1-6 cells pre-treated with a mixture of oleic acid/palmitic acid (2:1, M/M) for 24 h and then treated with 1 μM sitagliptin for 24 h. (**B**) Quantification of miR-200b and miR-200c in the livers of HFD-fed mice treated with sitagliptin. (**C**) Oil red O staining and H&E staining of the livers of HFD-fed mice treated with 3 mg/kg/day sitagliptin via i.g. for 8 weeks. (**D**) Measurement of hepatic triglyceride content in HFD-fed mice treated with sitagliptin. (**E**) Western blot analysis showing the expression of SREBP1 in the livers of sitagliptin-treated mice fed a HFD. (**F**, **G**) Western blot analysis showed that inhibition of miR-200b and miR-200c could partially rescue sitagliptin-induced reduced levels of SREBP1 and FAS, and decreased contents of intracellular triglyceride in Hep1-6 cells. The data represent the mean ± SEM, *n* = 6 mice or *n* = 3 independent experiments. *P < 0.05 and **P < 0.01 versus the control; & *P* < 0.05 versus NC+Sig. The bar represents 25 μm.

Studies have suggested that sitagliptin reduces hepatic lipid accumulation [26, 27]. However, the specific mechanism has not been fully elucidated. In the present study, Hep1-6 cells were pre-treated with a mixture of oleic acid/palmitic acid (2:1, M/M) for 24 h and then treated with 1 μM sitagliptin for 24 h. Real-time reverse-transcription PCR indicated that sitagliptin rescued the oleic acid/palmitic acid–induced reduction of miR-200b and miR-200c expression in Hep1-6 cells (Figure 7A). The study was further extended to HFD-fed mice. Interestingly, miR-200b and miR-200c were enhanced in the livers of HFD-fed mice treated with sitagliptin (Figure 7B). Oil red O and H&E staining showed that hepatic accumulation was improved when HFD-fed mice were administered 3 mg/kg/day sitagliptin via i.g. for 8 weeks compared with the vehicle control (Figure 7C). Hepatic triglyceride contents (Figure 7D) and SREBP1 expression levels (Figure 7E) were also reduced in sitagliptin-treated, HFD-fed mice. These data indicated that an increase in miR-200b and miR-200c expression might be associated with the sitagliptin-induced reversal of hepatic lipid accumulation in HFD-fed mice.

To further assess the requirement of miR-200 in sitagliptin's effect, miR-200b and miR-200c were knocked down in the Hep1-6 cells pre-treated with a mixture of oleic acid/palmitic acid and treated with sitagliptin. We found that sitagliptin treatment significantly decreased the expression of SREBP1 and FAS. More importantly, we demonstrated that down-regulation of miR-200b and miR-200c could partially reverse sitagliptin-induced decreased levels of SREBP1 and FAS, and reduced contents of intracellular triglyceride in Hep1-6 cells (Figure 7F, 7G).

## DISCUSSION

NAFLD is widely recognized as the most common cause of liver damage. It is characterized by abnormal fat accumulation in the liver and may eventually progress to nonalcoholic steatohepatitis (NASH) with progressive fibrosis [28]. Although great advances have been made in elucidating the pathogenesis of NAFLD, the underlying mechanism is still largely unknown. Emerging evidence suggests that miRNAs are involved in the signaling pathways of NAFLD. Previously, we examined miRNA expression in the livers of db/db mice and found that the expression of the miR-200 family members miR-200a, miR-200b and miR-200c was reduced in response to hepatic insulin resistance [23]. The miR-200 family has been extensively studied in different tumors [29–31]. Researchers demonstrated that the miR-200 family influences cancer development and progression by targeting various important signaling factors. However, few studies have been conducted on metabolic disorders. Our previous study demonstrated that the down-regulation of miR-200s following treatment with IL-6 could reduce the activation of the PI3K/AKT/GSK signaling pathway by targeting FOG2 [23]. In this study, we found that only miR-200b and miR-200c were decreased in the livers of NAFLD patients and mice fed a HFD. Additional experiments conducted in Hep1-6 and NCTC1469 cells revealed that the reduced expression of miR-200b and miR-200c led to abnormal lipid accumulation in hepatocytes, whereas the up-regulation of miR-200b and miR-200c impaired lipid accumulation and the expression of lipogenic proteins such as SREBP1 and FAS. These results indicate that miR-200b and miR-200c suppressed hepatic lipogenesis.

In this manuscript, we identified *jun* as a target gene of miR-200b and miR-200c. Bioinformatics predictions revealed that there were conserved miR-200b and miR-200c binding sites in the 3′UTR of *jun*. The presence of these sites was validated using a luciferase reporter assay. JUN is correlated with a variety of biological processes including cell survival, cell proliferation, apoptosis and development [24, 33]. Through its transcriptional activity, JUN controls the expression of numerous genes involved in a variety of physiological processes. As a member of the basic leucine zipper motif (bZIP) family of proteins, JUN contains a basic DNA-binding domain and a leucine zipper region [34, 35]. JUN–protein interactions comprise a complex regulatory network that functions in a cell type–sepcific manner [36]. In the present study, we identified two putative JUN sites TGCGTCA and TGACTCA [37] in the promoter region of murine *srebp1*, raising the possibility that JUN may bind to the *srebp1* promoter. The results of a luciferase reporter assay showed that the knockdown of *jun* significantly reduced the relative luciferase activity of the *srebp1* promoter. Furthermore, the suppression of *jun gene* obviously reduced the expression of SREBP1, indicating that JUN activates SREBP1 expression.

Sitagliptin, a dipeptidyl peptidase (DPP)-4 inhibitor, was found to maintain glucose homeostasis by reducing the inactivation of incretin hormones such as glucagon-like peptide-1 (GLP-1) [40]. *In vitro* studies demonstrated that GLP-1 could enhance the activation of adenosine monophosphate kinase and peroxisome proliferator-activated receptor-α, thereby inhibiting lipogenesis and increasing lipid oxidation, respectively [41, 42]. In this study, we provide preliminary evidence that the elevated expression of miR-200b and miR-200c might contribute to the sitagliptin-induced reduction of hepatic lipid accumulation in HFD-fed mice. The treatment of HFD-fed mice with sitagliptin significantly impaired hepatic lipid accumulation and reduced the expression of SREBP1. Interestingly, the levels of miR-200b and miR-200c were increased in the livers of HFD-fed mice treated with sitagliptin. Moreover, knock down of miR-200b and miR-200c could partially rescue sitagliptin-induced reduced levels of SREBP1 and FAS, and decreased contents of intracellular triglyceride in Hep1-6 cells

In conclusion, our findings may shed light on the specific molecular mechanism by which miR-200b and miR-200c are correlated with hepatic lipid metabolism. The reduced expression of miR-200b and miR-200c contributes to abnormal hepatic lipid accumulation by stimulating JUN expression and activating the transcription of *srebp1*.

## MATERIALS AND METHODS

### Experimental animals

Five-week-old male C57BL/6J mice were purchased from the Peking University Health Science Center. The C57BL/6J mice were fed a standard chow diet or a high-fat diet (HFD, D12451, 45% kcal from fat, Research Diet, USA, http://www.researchdiets.com/opensource-diets/stock-diets) for 10 weeks starting at approximately 5 weeks of age. The mice were housed in a temperature- (20–24°C) and humidity-controlled (45–55%) environment with a 12 h/12 h light/dark cycle. Ten weeks after the initiation of the HFD, the C57BL/6J mice were treated with 3 mg/kg/day sitagliptin via i.g. for 8 weeks. These animals were sacrificed, and liver tissues were collected for further analysis.

All of the mouse procedures were approved by the Animal Ethics Committee at the Beijing Hospital.

### Human liver specimens

The human liver biopsies and clinical procedures were performed with patient consent within the diagnostic workup of NAFLD. A total of 11 patients with NAFLD and 11 healthy control subjects were included in the study. Physical examinations, biochemical measurements, and body mass index (BMI) calculations were performed on all of the study participants. The application for patient-derived materials was approved by the Research Ethics Committee of Beijing You-An Hospital, and written consent was obtained from all of the patients.

### Cell culture and treatment

The NCTC1469 murine liver cell line and the Hep1-6 hepatic cancer cell line were purchased from American Type Culture Collection. Both of the cell lines were cultured in low-glucose Dulbecco's modified Eagle medium (DMEM) (Invitrogen, Carlsbad, California, USA) supplemented with 10% horse serum (Hyclone, Logan, Utah, USA) or 10% fetal bovine serum (Gibico, Grand Island, New York, USA), 100 units/ml penicillin (Invitrogen, Carlsbad, California, USA) and 0.1 mg/ml streptomycin (Hyclone, Logan, Utah, USA) at 37°C with humidified air and 5% CO_2_. Sitagliptin (Sigma-Aldrich, St. Louis, Missouri, USA) was used at a final concentration of 1 μM.

### Oleic acid/palmitic acid (O/P) treatment

0.25 M oleic acid (Sigma-Aldrich, St. Louis, Missouri, USA) and palmitic acid (Sigma-Aldrich, St. Louis, Missouri, USA) were dissolved in 100% ethyl alcohol. Before use, the 0.25 M oleic acid, the palmitic acid stock and 5% BSA were incubated in a 60°C water bath for 10 min. Then, 640 μl of the oleic acid or 320 μl of the palmitic acid stock were added drop-wise to 20 ml of 5% BSA to obtain 8 mM oleic acid and 4 mM palmitic acid, respectively. Before the experiments commenced, the 8 mM oleic acid and the 4 mM palmitic acid were incubated in a 60°C water bath for 10 min. Equal volumes of oleic acid and palmitic acid were mixed together. 300 μM oleic acid/palmitic acid mixture (2:1, M/M) was used to treat Hep1-6 and NCTC1469 cells for 24 h.

### RNA isolation and real-time reverse-transcription PCR

The miRNAs were isolated using TRIzol reagent (Invitrogen, Carlsbad, California, USA) according to the manufacturer's instructions. To detect and quantify the miRNAs, stem-loop reverse-transcription polymerase chain reaction (RT-PCR) was performed as previously reported [44]. The specific primers used in this study were described previously [23].

### Western blot analysis

The cells or liver tissues were lysed with RIPA buffer (Solarbio, Beijing, China). Fifteen μg of protein was separated by 10% SDS-PAGE and further transferred onto a PVDF membrane (Millipore, Boston, Massachusetts, USA). The membrane was blocked with 8% non-fat dry milk and incubated with primary antibodies at 4°C overnight. The specific primary antibodies used in this study were anti-SREBP1 (sc-366) (Santa Cruz, Dallas, Texas, USA), anti-FAS (#3180) (Cell Signaling, Boston, Massachusetts, USA), anti-β-actin (#3700) (Cell Signaling, Boston, Massachusetts, USA) and anti-JUN (#9165) (Cell Signaling, Boston, Massachusetts, USA). After washing three times with PBST, the membrane was incubated with the HRP-conjugated anti-rabbit or anti-mouse secondary antibody (Zhongshanjinqiao, Beijing, China. 1:5000) for 2 h at room temperature. Immunodetection was conducted using the ECL Plus detection system (Millpore, Boston, Massachusetts, USA) according to the manufacturer's instructions.

### Construction of adenovirus vectors

The adenovirus vector containing *jun* (Ad-*jun*, VH836318) and the control vector (Ad-NC, cp0001) were purchased from ViGene BioSciences (Rockville, Maryland, USA). And the adenovirus vector expressing mimics of miR-200b or miR-200c was constructed by Genechem (Shanghai, China).

### Chromatin immunoprecipitation (ChIP)

The Chromatin Immunoprecipitation Assay Kit was purchased from Millipore (Boston, Massachusetts, USA). Briefly, the nuclei extracted from cells transfected with Ad-JUN/NC or si-JUN/NC sonicated into 200–1000 bp. Precleared chromatin was immunoprecipitated with anti-JUN and normal IgG antibodies according to the manufacturer's instructions. Immunocomplexes were added into 50 μl of protein A/G-Sepharose beads and purified with Qiaquick (QIAGEN, Duesseldorf, German) PCR purification columns. The precipitated DNA was amplified with *srebp1*-specific primers. The primers specific to the JUN binding sites on the *srebp1* promoter were 5′-GTACCCTCAGGTCATACTGC-3′ and 5′-AAGCTCTCTGGATTGCCTT-3′.

### Oil Red O staining

Cells were treated with a mixture of oleic acid/palmitic acid (O/P, 2:1, M/M) for 24 h and washed three times with PBS (5 min each). The cells were then fixed with 4% paraformaldehyde for 10 min at room temperature. After dehydration with 100% 1,2-propanediol for 5 s, the cells were stained with pre-warmed Oil Red O for 30 min at 37°C and washed with 85% 1,2-propanediol. The cells were washed with distilled water and observed under a microscope.

### Triglyceride measurement

The triglycerides in liver tissues or cells were extracted according to a modified method from Folch *et al.* [45]. Briefly, snap-frozen liver tissues (approximately 20 mg) were homogenized in 5 ml of 1 M NaOH and extracted with 5 ml of a chloroform/methanol (v/v = 2:1) solution. The organic layer was dried under nitrogen gas and resolubilized in 1 ml of chloroform containing 1% Triton X-100. The extract was dried again and resuspended in 1 ml of water. The triglyceride concentration was measured using a triglyceride enzymatic assay kit (ShenSuoYouFu Medical Diagnostic Products Co., Ltd., Shanghai, China).

### Histological analysis of tissues

The liver samples were fixed in O.C.T. compound (Tissue-Tek, Japan) and sectioned at a thickness of 8 μm. The slides were stained with hematoxylin & eosin (H&E) or with Oil Red O as previously described [46, 47].

### Luciferase reporter assay

For the luciferase assay, the *jun* 3′UTR sequence was cloned into the pmirGLO plasmid as previously described [44]. The PCR cycling conditions were as follows: (1) a hot start step at 95°C for 10 min; (2) 40 cycles at 95°C for 15 s and 55°C for 45 s and (3) 72°C for 30 s. The mutant was cloned using the Fast Mutagenesis System (TransGen Biotech, Beijing, China). The primers used to detect *jun* were 5′-CGAGCTCGGCCAAGGGTACACAAGATGG-3′ and 5′-CCGCTCGAGCGGGGTCCCTGCTTTGAGAA TCA-3. *jun* -Mut-Forward: 5′-TTCGATCTCATTCAGTC GGAAAGGGGGG-3′; *jun*-Mut-Reverse: 5′- CCGACTGA ATGAGATCGAATGTTAGGTCCA-3′.

Before the luciferase reporter assay was carried out, 5 × 10^4^ cells in 500 μl of L-DMEM were seeded in each well of a 24-well plate and cultured for 18 h. The cells were transfected with the modified firefly luciferase vector (500 ng/μl) mixed with the Vigofect transfection reagent (Beijing, China) according to the manufacturer's instructions. After 48 h, the luciferase activities were measured using the dual-luciferase reporter assay system (Promega, Madison, Wisconsin, USA). Renilla luciferase activity was used as a normalization control.

### Promoter reporter analysis

The PGL3 promoter vector and the PRL-TK vector were purchased from Promega Corporation. The promoter region of *srebp1* was amplified from the genomic DNA of Hep1-6 cells. The PGL3 promoter vector and the amplified fragments were digested with *Xho*I/KpnI and purified by gel electrophoresis. The digested fragment was then inserted into the PGL3 vector up-stream of the SV40 promoter. HEK293T cells were co-transfected with the PGL3 plasmids and the PRL-TK vector using the VigoFect Transfection Reagent (Beijing, China). The cells were harvested and lysed 48 h post-transfection. The relative light units (RLU) were determined using the Dual-luciferase reporter assay system (Promega, Madison, Wisconsin, USA) according to the standard protocols. Normalized luciferase data (firefly/renilla) was compared with the empty pGL3-promoter vector.

The RLU were determined using the dual-luciferase reporter assay system (Promega, Madison, Wisconsin, USA) according to the standard protocol. The primers for amplification were as follows: *srebp1*-F: 5′-GGGTACCCTTCTCTGCTCAACGAGGT CA-3′, *srebp1*-R: 5′-CCTCGAGGTCTTTACCCTGTGC GGAA-3, *srebp1*-S1-F: 5′-GGGTACCCCTCAACGAGG TCAAAGA-3′, *srebp1*-S1-R: 5′-CCTCGAGGTACTCTG GTTTTGGTCAC-3′, *srebp1*-S2-F: 5′-GGGTACCCTTC TCTGCTCAACGAGGTCA-3′ and *srebp1*-S1-R: 5′-CCTC GAGGTCTCAGCCCGACCGACAG-3′. The restriction sites for KpI and XhoI are underlined.

### Cell transfections

The miR mimics, inhibitors and negative control (NC) were purchased from Genepharma (Shanghai, China), as were the siRNA-JUN and the non-specific siRNA (NC). The miR and siRNA transfections were performed using the HiPerfect transfection reagent (QIAGEN) as previously described [44]. Briefly, 1 × 10^6^ cells in 2 ml of L-DMEM culture medium supplemented with serum and antibiotics were seeded in 6-well plates. At the same time, the miR mimics, inhibitors or NC were mixed with HiPerfect transfection reagent (QIAGEN, Duesseldorf, Germany) and incubated at room temperature for 10 min. The NCTC1469 and Hep1-6 cells were incubated with the complex for 48 h.

### Statistics

The data represent the mean ± standard error of the mean (SEM). Differences were analyzed by Student's *t*-test, and statistical significance was set at *P* < 0.05.
