# Correction: Reduced miR-200b and miR-200c expression contributes to abnormal hepatic lipid accumulation by stimulating JUN expression and activating the transcription of srebp1

**DOI:** 10.18632/oncotarget.28562

**Published:** 2024-05-17

**Authors:** Jun Guo, Weiwei Fang, Libo Sun, Yonggang Lu, Lin Dou, Xiuqing Huang, Mingxiao Sun, Cheng Pang, Jing Qu, Guanghui Liu, Jian Li

**Affiliations:** ^1^The Key Laboratory of Geriatrics, Beijing Hospital and Beijing Institute of Geriatrics, Ministry of Health, Beijing 100730, China; ^2^Graduate School of Peking Union Medical College and Chinese Academy of Medical Sciences, Beijing 100730, China; ^3^National Laboratory of Biomacromolecules, Institute of Biophysics, University of Chinese Academy of Sciences, Chinese Academy of Sciences, Beijing 100101, China; ^4^Department of Hepatobiliay Surgery and You-An Liver Transplantation Center, Beijing You-An Hospital, Capital Medical University, Beijing 100069, China; ^5^State Key Laboratory of Stem Cell and Reproductive Biology, Institute of Zoology, Chinese Academy of Sciences, Beijing 100101, China; ^6^University of Chinese Academy of Sciences, Beijing 100049, China; ^7^These authors contributed equally to this work

**This article has been corrected:** Due to errors during figure preparation, the actin bands in Figure 1C contain partial duplications of the actin bands in Figure 5D and 5E. Similarly, in Figure 3D, the SREBP1 band contains partial duplications of the JUN band in Figure 4D. The corrected versions of Figure 1C and Figure 3D, obtained using the original data, are presented below. The authors declare that these corrections do not change the results or conclusions of this paper.

Original article: Oncotarget. 2016; 7:36207–36219. 36207-36219. https://doi.org/10.18632/oncotarget.9183

**Figure 1:**
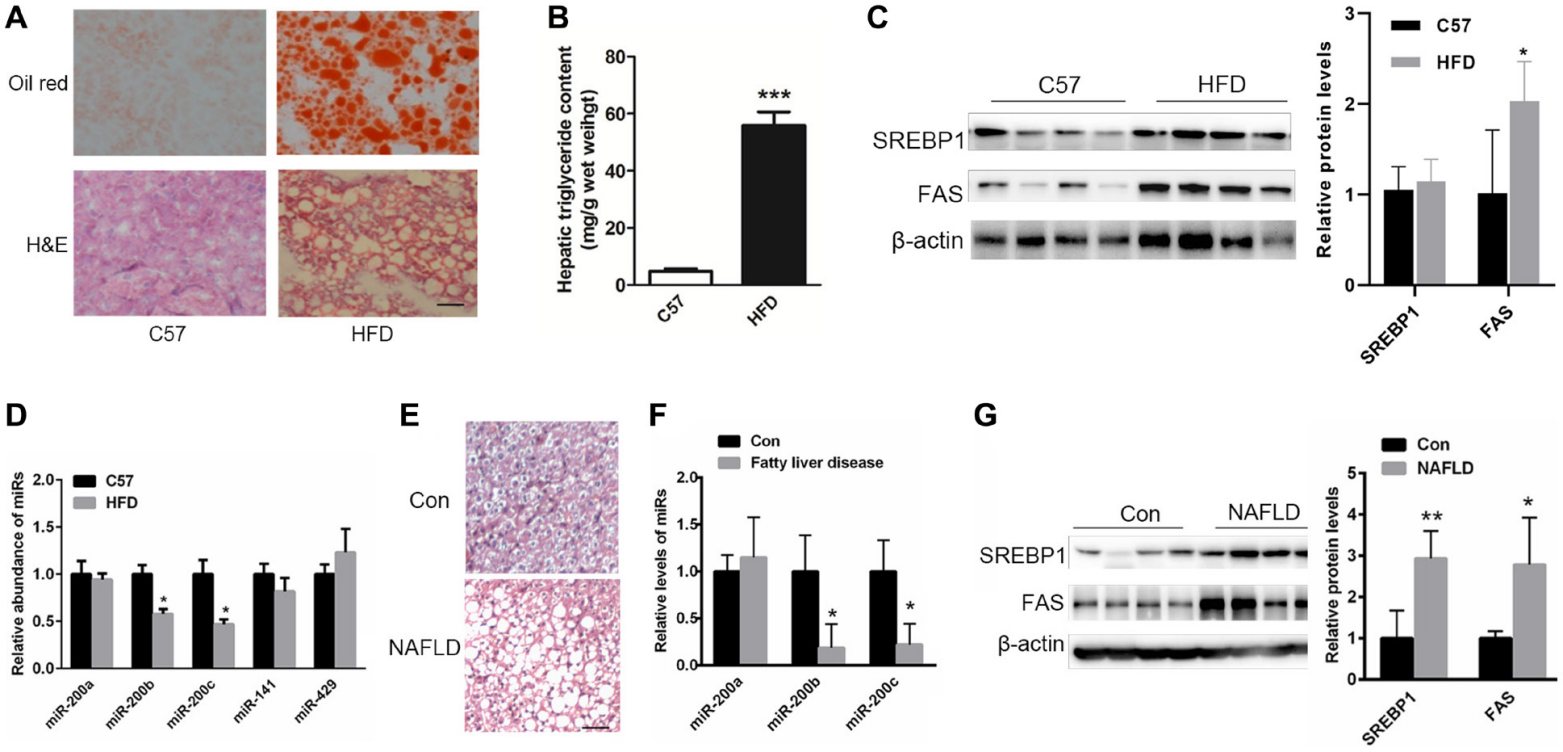
The levels of miR-200b and miR-200c are reduced in the steatotic livers of NAFLD patients and mice fed a HFD. (**A**) Oil red O and H&E staining of the livers of HFD-fed mice. (**B**) The measurement of triglyceride levels in the livers of HFD-fed mice. (**C**) Western blots showing the expression of the lipogenic proteins SREBP1 and FAS. (**D**, **F**) Real-time reverse-transcription PCR showing the relative expression patterns of miR-200 family members including miR-200b, miR-200c, miR-200a, miR-141 and miR-429 in the steatotic livers of HFD-fed mice (*n* = 5) or in the livers of NAFLD patients and healthy subjects (*n* = 11). (**E**) H&E staining of the livers of NAFLD patients. (**G**) Western blots showing the expression of SREBP1 and FAS in the livers of NAFLD patients. The data represent the mean ± SEM. ^*^ *P* < 0.05 and ^**^ *P* < 0.01 versus the control. The bar represents 25 μm.

**Figure 3:**
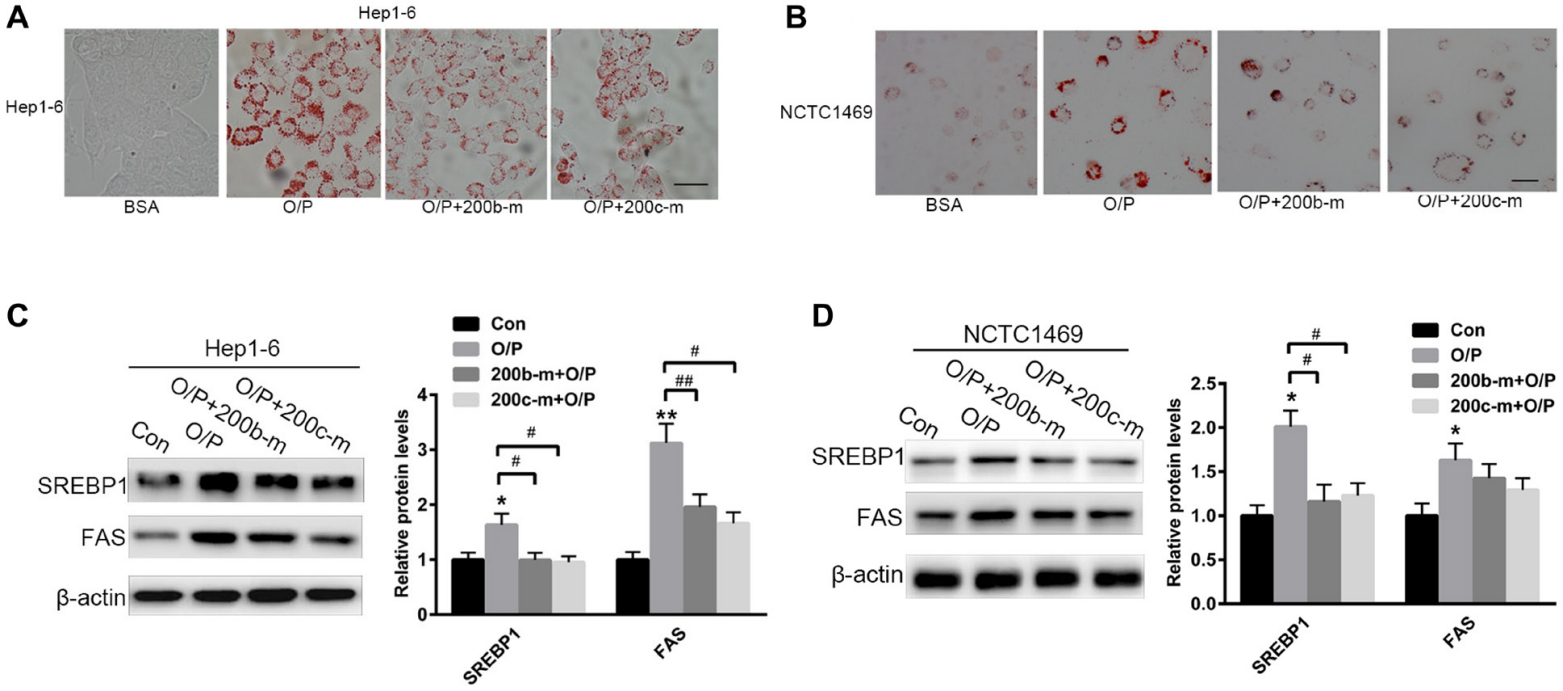
The over-expression of miR-200b and miR-200c reverses oleic acid/palmitic acid-induced lipid accumulation in hepatocytes. (**A**, **B**) Oil red O staining of Hep1-6 and NCTC1469 cells pre-treated with a mixture of oleic acid/palmitic acid (2:1, M/M) for 24 h. (**C**, **D**) Western blots showing the expression of SREBP1 and FAS in Hep1-6 and NCTC1469 cells pre-treated with a mixture of oleic acid/palmitic acid (2:1, M/M) for 24 h and then transfected with miR-200b and miR-200c mimics. The data represent the mean ± SEM of three independent experiments. ^*^ *P* < 0.05 and ^**^ *P* < 0.01 versus the control; ^#^ *P* < 0.05 and ^##^ *P* < 0.01 versus O/P. The bar represents 10 μm.

